# Hurricanes, industrial animal operations, and acute gastrointestinal illness in North Carolina, USA

**DOI:** 10.1088/2752-5309/ad9ecf

**Published:** 2024-12-27

**Authors:** Arbor J L Quist, Mike Dolan Fliss, David B Richardson, Paul L Delamater, Lawrence S Engel

**Affiliations:** 1 Division of Epidemiology, College of Public Health, The Ohio State University, Columbus, OH 43210, United States of America; 2 Department of Epidemiology, Gillings School of Global Public Health, University of North Carolina, Chapel Hill, NC 27599, United States of America; 3 Injury Prevention Research Center, University of North Carolina, Chapel Hill, NC 27514, United States of America; 4 Department of Geography, University of North Carolina, Chapel Hill, NC 27514, United States of America

**Keywords:** hurricanes, CAFOs, swine, hog production, gastrointestinal illness, environmental justice, flooding

## Abstract

North Carolina (NC) ranks third among US states in both hog production and hurricanes. NC’s hogs are housed in concentrated animal feeding operations (CAFOs) in the eastern, hurricane-prone part of the state. Hurricanes can inundate hog waste lagoons, transporting fecal bacteria that may cause acute gastrointestinal illness (AGI). While CAFOs and hurricanes have separately been associated with AGI, few epidemiological studies have examined the joint effect of hurricanes and CAFOs. We examined the impacts of Hurricanes Matthew (2016) and Florence (2018) on the occurrence of post-storm AGI in areas with varying numbers of hog and poultry CAFOs. We used ZIP code-level disease surveillance data, 2016–2019, to calculate rates of AGI emergency department (ED) visits in NC. Using precipitation data, CAFO permit data, and interrupted time series methods, we assessed the change in AGI rate during the three weeks after Matthew and Florence in ZIP codes with heavy rain (>75th percentile of storm precipitation) and 0, 1–10, and >10 hog CAFOs. The AGI ED rate in ZIP codes with heavy storm rain and >10 hog CAFOs increased 15% (RR = 1.15, 95% CI: 1.04, 1.27) during the three weeks after Hurricane Florence, although there was little increase after Hurricane Matthew (RR = 1.05, 95% CI = 0.86, 1.24). The AGI ED rates in ZIP codes with heavy storm rain and no hog CAFOs exhibited no increase during these post-hurricane periods (Matthew: RR = 0.97, 95% CI: 0.80, 1.14; Florence: RR = 1.01, 95% CI: 0.89, 1.13). We also observed an increase in AGI ED rate in areas with both >10 hog CAFOs and >10 poultry CAFOs. Areas with heavy hurricane precipitation and many CAFOs had a higher proportion of Black, American Indian, and Hispanic residents and lower annual household incomes than the state averages. Heavy hurricane precipitation in areas with CAFOs may increase AGI rates, disproportionately affecting people of color in NC.

## 1. Introduction

Hurricanes are often destructive and can lead to acute and longer-term adverse health outcomes. Beyond immediate traumatic injuries, hurricanes can aggravate existing environmental health issues, such as when heavy precipitation and flooding spread pathogens and chemicals from flooded hazardous waste sites, oil refineries, animal manure ponds, or other industrial sites [1–5]. Environmental contamination exacerbated by hurricanes varies by region. As North Carolina (NC) is the third top hog producer in the United States (US) with 9 million hogs and also the third most hurricane-prone US state [6, 7], hurricanes that strike NC may inundate hog manure ponds and result in contamination of nearby waterways [8]. Most NC hogs are housed, with thousands in a single building, at large concentrated animal feeding operations (CAFOs) in the eastern, hurricane-prone region of the state [9]. NC industrial animal operations produce over nine billion gallons of fecal waste annually [10]. Liquid fecal waste from hogs is collected in uncovered pits, or lagoons, which are regularly sprayed onto neighboring fields [8, 11]. During heavy rain and hurricanes, fecal bacteria from manure-applied fields or from flooded lagoons may be transported from CAFOs into nearby waterways [8, 11]. Surface water near hog and poultry CAFOs has been found to have elevated levels of fecal indicator bacteria, nitrogen, and phosphorus [12–14]. Contact with pathogens from hog manure (e.g. *Escherichia coli, Salmonella, Campylobacter, Yersinia enterocolitica, Cryptosporidium, Giardia*) may cause diarrhea, vomiting, nausea, or other gastrointestinal distress in humans, collectively referred to as acute gastrointestinal illness (AGI) [15, 16]. AGI is painful and can be detrimental to health, especially in young children and older adults [17]. Approximately 2330 000 waterborne enteric illnesses occurred in 2014 in the US, which incurred about $160 million in direct healthcare costs [18]. Although news reporters regularly discuss the dangers of flooded hog CAFOs when large hurricanes strike NC, very few studies have examined the effect of flooded hog CAFOs in NC on AGI [19–21].

Communities near hog CAFOs have reported various health problems, including diarrhea, headaches, methicillin-resistant *Staphylococcus aureus*-related infections, impaired quality of life, and eye, nose, and throat irritation [22]. Many residents near CAFOs use private wells, which have a higher risk of contamination than community water supplies [16, 23]. Hog CAFOs are densely concentrated in rural, eastern NC counties that typically have reduced healthcare access, have a higher percentage of people of color than the state average, and are also home to other detrimental industrial exposures like poultry CAFOs and landfills [24–27]. CAFO exposure in NC is an environmental justice issue. Multiple studies have found that vulnerable subpopulations have disparate exposure to CAFOs, including Black and Hispanic residents in Wisconsin and low-income communities in Delaware and North Carolina [28–31]. These vulnerable populations living near CAFOs may also be particularly vulnerable during natural disasters.

Hurricane Matthew (2016) and Hurricane Florence (2018) were the two largest hurricanes to strike NC in the past decade and cost the state $1.5 billion and $22 billion, respectively [32, 33]. Hurricane Florence drenched NC with 8 trillion gallons of water in one week, making it the wettest hurricane on record in the state [34]. Hurricane Matthew caused at least 14 hog manure lagoons to flood and 2 lagoons to breach [35], and at least 110 hog manure lagoons were breached or inundated in NC due to Hurricane Florence [36]. Hurricane flooding in North Carolina has led to elevated fecal coliform levels, high nutrient concentrations, and severe dissolved oxygen deficits in surface water, some of these elevations may be due to CAFOs and sewage treatment plants [37–40].

This paper examines the combined effect of hurricane precipitation and hog CAFO exposure on AGI in NC and assesses this effect across two different hurricanes—Hurricanes Matthew and Florence. Previous studies have found hurricanes and high hog CAFO exposure to be associated with increased AGI rates [27, 41], but this is the first study to examine how the rates of AGI emergency department (ED) visits in NC change after hurricanes in areas with heavy hurricane precipitation and varying exposure to hog CAFOs. Understanding the connection between flooding, hog CAFOs, and health is important in developing appropriate interventions, especially as climate change models predict that NC will continue to see an increase in heavy precipitation events [42, 43].

## 2. Methods

### 2.1. Study population

The study population comprises of NC residents who lived in areas that received heavy precipitation during Hurricanes Matthew or Florence, including residents who lived near many or no hog CAFOs. Cases include NC residents who visited a NC ED in 2016–2019 and had an AGI-related diagnosis code. The finest geographic resolution ED data was the ZIP code level; thus, all analyses were conducted at this level.

### 2.2. Rainfall exposure

Hurricane Matthew struck NC on 8 October 2016, and Hurricane Florence hit NC on 14 September 2018. We examined the change in AGI ED rate during the three weeks after the hurricanes by using 2016–2019 data trends to estimate the predicted AGI ED visit rate had the events not occurred. We were interested in a three-week post-hurricane period because there is likely a lag between water contamination and human exposure to contaminated water, because flooding from Hurricane Florence lasted a week or more in some areas, and because the AGI-causing pathogens in floodwater have up to a two-week incubation period [41]. We obtained daily precipitation data as 4 km-by-4 km raster data from the Parameter-elevation Regressions on Independent Slopes Model Climate Group [44]. We then subsampled this data into 1 km raster data and used the 1 km centroids to aggregate the precipitation data to the NC ZIP code polygons. We assigned ZIP codes the daily maximum precipitation recorded in the ZIP code. For each ZIP code, we summed the daily maximum precipitation during the week of Hurricanes Matthew (08 October 2016–14 October 2016) and Florence (14 September 2018–20 September 2018) to capture the total hurricane precipitation by area for each storm. ZIP codes in the top quartile of hurricane precipitation (Matthew: >9 inches/229.67 mm; Florence: >12.8 in/325.19 mm) were categorized as severely affected by the hurricane (‘heavy storm precipitation’).

### 2.3. CAFO exposure

**Figure 1.**
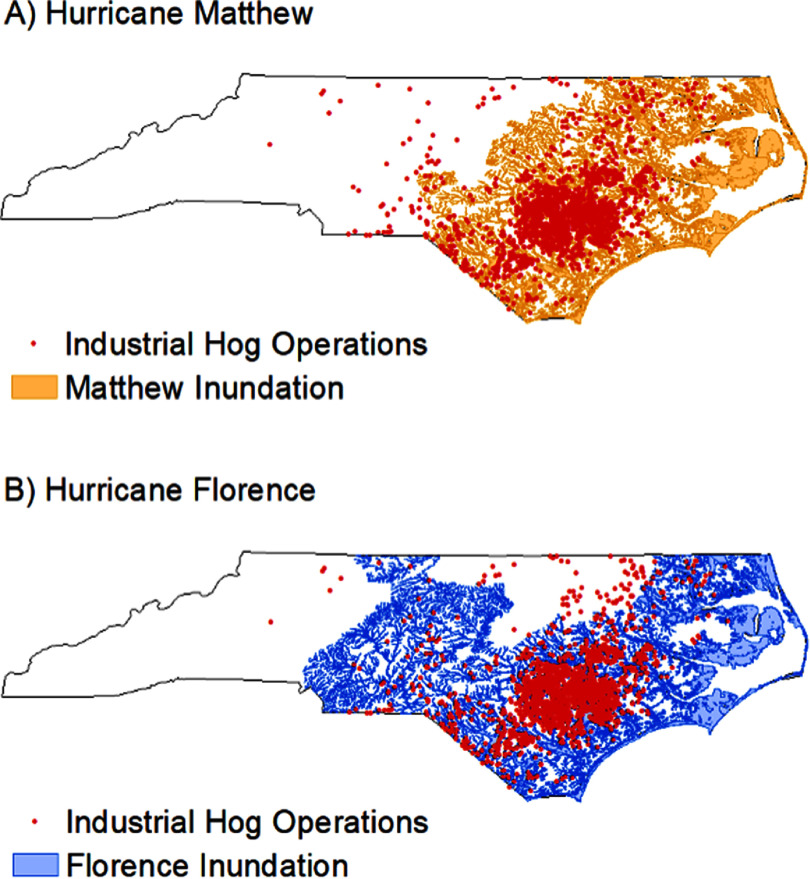
Location of industrial hog operations and hurricane flood inundation from (A) Hurricane Matthew and (B) Hurricane Florence (industrial hog operation locations from NC Department of Environmental Quality’s 2019 swine permit data and Hurricanes Matthew and Florence flood inundation data from NC Department of Public Safety).

We used 2019 swine permit data from the NC Department of Environmental Quality (DEQ), which included the location, number of animals, and type/life stage of animals of each permitted animal facility (hog CAFO locations shown in figure 1). We counted the number of hog CAFOs contained within each ZIP code or a half mile of each ZIP code’s geographical boundary. We categorized areas with no hog CAFOs as ZIP codes that neither contain a hog CAFO nor have any hog CAFOs within a half mile of the ZIP code border. We categorized ZIP codes with hog CAFOs into low hog CAFO-exposed ZIP codes (1–10 hog CAFOs within the ZIP code or within a half mile of the border) and high hog CAFO-exposed ZIP codes (>10 hog CAFOs).

Poultry CAFOs are often co-located near hog CAFOs in NC, and exposure to pathogens that may be found in poultry waste can also lead to AGI. Our main analyses focused on hog CAFOs because hog CAFOs produce mostly liquid waste collected in uncovered lagoons that can flood while poultry CAFOs produce mostly dry waste; however, we also examined poultry CAFOs and the co-location of both poultry and hog CAFOs. We obtained data on poultry CAFO locations from the Environmental Working Group and Waterkeepers Alliance. They identified these locations using high-resolution satellite data and aerial photographs; they also estimated the number of birds at each facility using the National Agriculture Imagery Program (2008, 2012, 2016, 2019) as well as the 2017 Census of Agriculture from the United States National Agricultural Statistics Service [10]. We developed similar categories for poultry CAFOs as we did for hog CAFOs. ZIP codes with no poultry CAFOs within the ZIP code or a half a mile of the border were categorized as 0 poultry CAFOs. ZIP codes with poultry CAFOs were categorized into 1–10 poultry CAFOs and >10 poultry CAFOs within the ZIP code or a half a mile of the border. We also developed categories of ZIP codes with heavy storm precipitation and >10 hog CAFOs and >10 poultry CAFOs, heavy storm precipitation and no hog CAFOs or poultry CAFOs, and a middle category for the ZIP codes with heavy storm precipitation and some CAFOs.

### 2.4. Outcome

AGI was measured using data from the NC Disease Event Tracking and Epidemiologic Collection Tool (NC DETECT), a public health surveillance system of civilian ED visits in NC. AGI rates for 2016–2019 were calculated at the ZIP code level. We used diagnostic codes (International Classification of Diseases, Tenth Revision; ICD-10) to classify intestinal infectious illness (A00–A09), unspecified noninfectious gastroenteritis and colitis (K52.3, K52.89, K52.9), diarrhea (R19.7), and nausea and vomiting (R11.10-R11.12) as AGI ED visits. Similar diagnosis codes have been used in other studies of flooding and AGI [23, 41, 45, 46]. Our analyses examined all-cause AGI rates because specific pathogens are seldom tested for and are rarely included in hospital discharge reports.

### 2.5. Statistical methods

Data on the total number of residents and other demographics were available at the block group-level from the 2017 American Community Survey (ACS). We assigned these values to the centroids of each 2010 Census block based on the proportion of the block group population within that block and then aggregated these block centroid data to create ZIP code-level population estimates. We also used the 2018 CDC/ATSDR social vulnerability index (SVI) for NC to examine the other social and environmental exposures and vulnerabilities that residents living near hog CAFOs and hurricane flooding face [47]. The SVI assesses Census tract-level vulnerability in terms of socioeconomic status (SES), household composition and disability, minority status and language, and housing type and transportation. The SVI ranges from 0 to 1, with 1 being the most vulnerable. We attributed the tract-level SVI scores to the ZIP code level by taking the mean of the scores inside each NC ZIP code.

**Figure 2.**
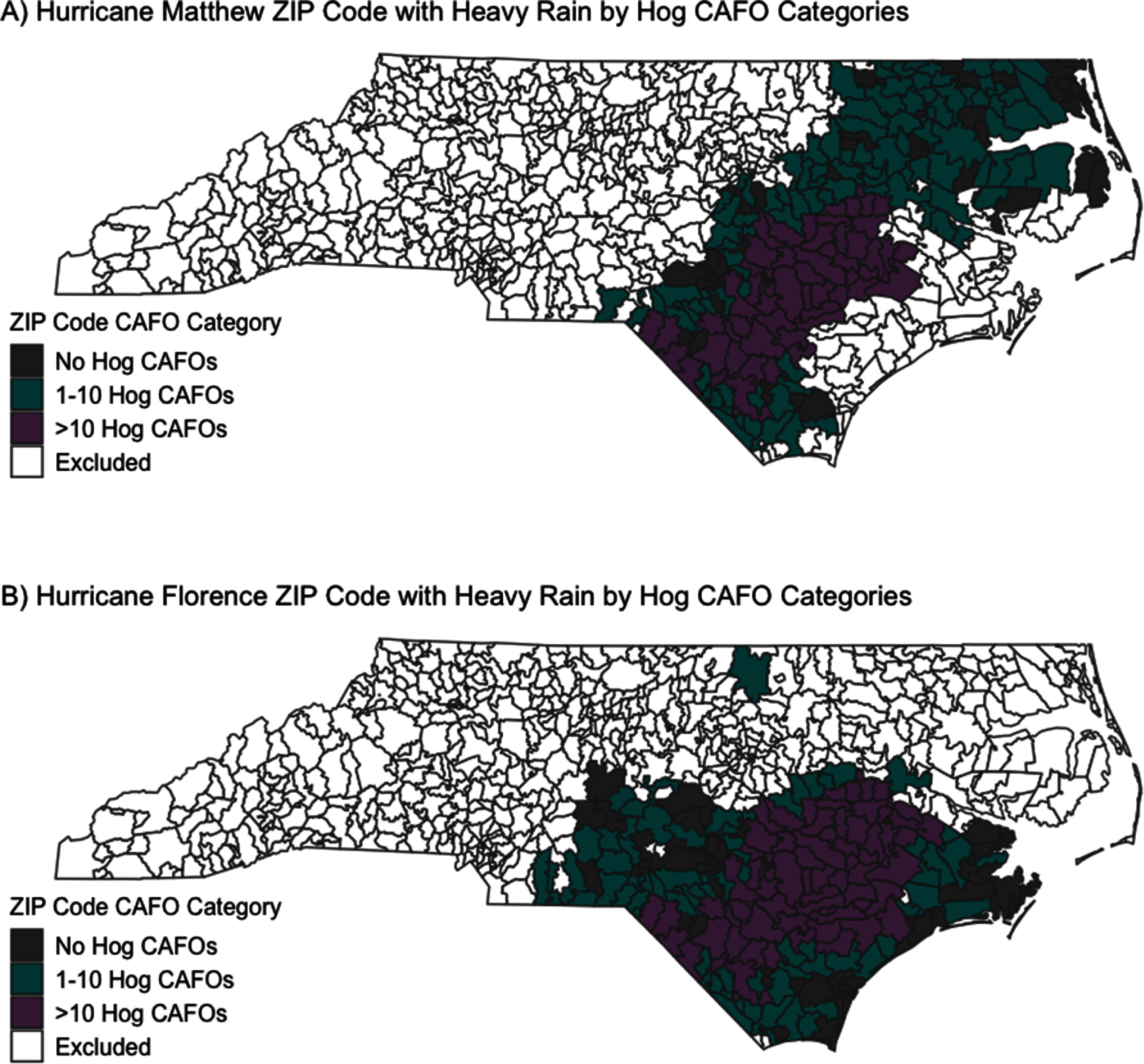
ZIP codes with heavy hurricane precipitation (>75th percentile of total hurricane week precipitation) and hog CAFO category for (A) Hurricane Matthew and (B) Hurricane Florence.

We first described the demographics of residents living in ZIP codes with heavy hurricane rain and the various hog CAFO categories, as well as statewide, to assess exposure disparities. We assessed the change in AGI ED rate during the three weeks after Hurricanes Matthew and Florence in areas with heavy storm precipitation and 0 hog CAFOs, heavy storm precipitation and 1–10 hog CAFOs, and heavy storm precipitation and >10 hog CAFOs (see figure 2). We used interrupted time series, which allows every ZIP code to be compared to itself over time [48]. This method uses the daily AGI ED rate in each ZIP code (2016–2019) to predict the AGI ED rate after the hurricanes had there not been a hurricane. We modeled Hurricanes Matthew and Florence separately. Because of potential over-dispersion of the outcome, we used quasi-Poisson models; the regression model included indicator variables for the post-hurricane flood period and time-control variables for the day of week, month, and year, and an interaction between month and year. To estimate the change in population-based AGI ED visit *rate* after a hurricane, the yearly ZIP code population (derived from ACS data) was included as an offset in the model. Robust standard errors were used to calculate 95% confidence intervals (95% CI) using the *sandwich* package in R.

In sensitivity analyses, we also examined the change in AGI ED visit rate during the 1, 2, 4, and 5-week periods after the hurricanes. Additionally, we examined the change in AGI ED visit rate during the three weeks after the hurricanes in ZIP codes with heavy storm precipitation and >20 hog CAFOs. Because CAFOs have differing numbers of animals, we also conducted sensitivity analyses based on total number of hogs and birds in CAFOs within ZIP codes (0 hogs, 1–10 000 hogs, >10 000 hogs; 0 birds, 1–1000 000 birds, >1000 000 birds; 0 hogs and birds, 1–10 000 hogs and/or 1–10 000 000 birds, >10 000 hogs and >1000 000 birds). Because Hurricane Florence dropped substantially more rain than Hurricane Florence, we conducted a sensitivity analysis for Hurricane Matthew that only included ZIP codes that received >12.8 in/325.19 mm precipitation (Hurricane Florence’s heavy precipitation threshold) during the week of Hurricane Matthew.

We also conducted a sensitivity analysis that included sanitary sewer overflow (SSO) data, as hurricane precipitation can also cause sewer overflows, which can spread fecal pathogens and could lead to AGI. The SSO data was provided from the NC DEQ, Division of Water Resources. This data reported all reported SSO incidents from 2016 to 2018 by county and included the date and estimated total volume in gallons. In this sensitivity analysis, we conducted interrupted time series analyses while adjusting for SSOs within the county within the past two weeks (see SI). Lastly, we examined the change in rate of *all* ED visits (not just AGI visits) during the three weeks after the hurricanes to assess if our results were driven by ED usage patterns after storms or specific to AGI. All analyses were performed in R (Version 4.1.3) [49].

## 3. Results

There were a total of 2714 hog CAFOs in areas that received heavy rain (>75th percentile of storm precipitation) during Hurricane Matthew and 2964 hog CAFOs in areas that received heavy rain during Hurricane Florence. In ZIP codes with heavy storm rain and >10 hog CAFOs, there were 663 AGI ED visits during the 3 weeks after Hurricane Matthew and 1063 AGI ED visits during the 3 weeks after Hurricane Florence, with 670 AGI ED visits the 3 weeks before Matthew and 927 AGI ED visits the three weeks before Florence. ZIP codes that contained >10 hog CAFOs and received heavy rain during Hurricanes Matthew or Florence had a higher proportion of Black, American Indian, and Hispanic residents and uninsured residents than the state average (table 1). ZIP codes with heavy storm precipitation and >10 hog CAFOs also had lower household annual median incomes and higher poultry density than both the state average and areas with heavy storm precipitation and no hog CAFOs. Additionally, areas with heavy storm precipitation and >10 hog CAFOs were more vulnerable areas according to every SVI scale—SES, disability, minority status, transportation, and total vulnerability. ZIP codes with many hog CAFOs and heavy storm precipitation are more rural, geographically isolated, and have lower overall AGI ED visit rates than ZIP codes with heavy storm precipitation and no hog CAFOs (table 1).

**Table 1.** Characteristics of ZIP codes categorized by hog CAFO and heavy storm rain (>75th percentile of storm precipitation) status.

		Hurricane Matthew			Hurricane Florence		
	Statewide	0 hogs and heavy storm rain	1–10 hog CAFOs and heavy storm rain	>10 hog CAFOs and heavy storm rain	0 hogs and heavy storm rain	1–10 hog CAFOs and heavy storm rain	>10 hog CAFOs and heavy storm rain
Total Population^a^	10 207 847	2516 419	1393 041	623 927	3491 368	1316 797	715 320
American Indian (%)	117 943 (1.2)	30 280 (1.2)	21 255 (1.5)	34 208 (5.5)	33 600 (1.0)	21 180 (1.6)	34 479 (4.8)
Asian (%)	269 011 (2.6)	75 495 (3.0)	20 503 (1.5)	4721 (0.8)	115 470 (3.3)	21 465 (1.6)	4920 (0.7)
Black (%)	2157 353 (21.1)	716 040 (28.5)	406 438 (29.2)	175 311 (28.1)	821 111 (23.5)	358 474 (27.2)	196 785 (27.5)
Hispanic (%)	914 673 (9.0)	262 405 (10.4)	113 267 (8.1)	71 772 (11.5)	353 990 (10.1)	112 013 (8.5)	78 756 (11.0)
Pacific Islander (%)	6304 (0.1)	2527 (0.1)	824 (0.1)	290 (0)	2968 (0.1)	1002 (0.1)	304 (0)
White non-Hispanic (%)	6932 516 (67.9)	1501 131 (59.7)	842 665 (60.5)	361 375 (57.9)	2254 856 (64.6)	813 109 (61.7)	420 462 (58.8)
Other Race (%)	311 318 (3.0)	85 760 (3.4)	40 267 (2.9)	23 100 (3.7)	119 228 (3.4)	40 937 (3.1)	25 811 (3.6)
Uninsured (%)	1185 916 (11.6)	302 222 (12.0)	168 827 (12.1)	88 899 (14.2)	418 509 (12.0)	157 410 (12.0)	100 630 (14.1)
Age 65 and older (%)	151,3377 (14.8)	335 123 (13.3)	206 953 (14.9)	93 827 (15.0)	491 317 (14.1)	193 554 (14.7)	107 877 (15.1)
Annual Median Income ($)	48 095	51 419	42 527	37 929	50 214	43 978	38 333
Hog CAFOs	2489	0	300	1762	0	238	2083
Hogs (N)	877,3273	0	1085 740	6434 168	0	792 035	7400 431
Hog density (hogs/sqmi)	177	0	97	1126	0	92	1072
Poultry CAFOs	4808	216	879	1175	370	932	1396
Poultry (N)^b^	538 006 718	23 981 531	107 758 267	135 368 004	40 681 455	116 623 733	162 095 705
Poultry density (birds/sqmi)	10 827	4195	9599	23 700	4784	13 601	23 473
Isolation Distance^c^	7.2	6.3	7.9	7.9	6.1	7.6	8
AGI ED visits in study period	868 623	206 085	137 214	64 563	293 062	131 265	73 876
Total ED visits in study period	15 373 282	3546 091	1985 167	1269 754	4342 107	1949 052	1482 413
AGI rate per 1000 ED visits	56.5	58.1	69.1	50.8	67.5	67.3	49.8
SVI—Total^d^	0.54	0.60	0.67	0.79	0.54	0.66	0.77
SVI—SES	0.57	0.62	0.69	0.78	0.57	0.69	0.77
SVI—Disability	0.56	0.64	0.67	0.78	0.59	0.69	0.76
SVI—Minority	0.46	0.50	0.52	0.62	0.43	0.51	0.60
SVI—Transportation	0.51	0.52	0.57	0.66	0.51	0.56	0.64

^a^ Demographic data from 2017 American Community Survey.

^b^ Data on the location of poultry CAFOs and estimated number of birds at each CAFO was provided by the Environmental Working Group and Waterkeepers Alliance. They identified poultry facility locations with high-resolution satellite data and aerial photograph and estimated number of birds at each poultry CAFO using the NC Agricultural Chemical Manual and the US Department of Agriculture’s Ag Census. Statewide and Hurricane Florence estimates are from 2018 data; the Hurricane Matthew poultry estimates are from 2016 data [50].

^c^ Rurality was measured using a continuous geographic isolation scale that classifies ZIP codes according to their access to resources [51].

^d^ The 2018 CDC/ATSDR social vulnerability index (SVI) assesses vulnerability in terms of socioeconomic status (SES), household composition and disability, minority status and language, and housing [47]. The SVI ranges from 0 to 1, with 1 being the most vulnerable.

We observed an 15% increase in AGI ED visit rate (rate ratio [RR] = 1.15, 95% confidence interval [CI]: 1.04, 1.27) during the three weeks after Hurricane Florence among ZIP codes with >10 hog CAFOs and heavy storm precipitation compared to the expected AGI ED rate at this time based on 2016–2019 trends (table 2). We did not observe a substantial increase in AGI ED visit rate (RR = 1.05, 95% CI: 0.86, 1.24) during the three weeks after Hurricane Matthew in ZIP codes with >10 hog CAFOs and heavy Hurricane Matthew precipitation. We did not observe an increase in AGI ED visit rate in ZIP codes with heavy storm precipitation and no hog CAFOs or in ZIP codes with heavy storm precipitation and 1–10 hog CAFOs during the three weeks after Hurricanes Matthew or Florence (table 2).

**Table 2.** The change in AGI ED rate during the three weeks after Hurricanes Matthew and Florence in ZIP codes in top quartile of storm precipitation and varying numbers of hog and poultry CAFOs using interrupted time series.

Number of CAFOs	RR (95% CI)	Number of ZIP codes	Number of AGI cases post-storm	Storm rain mean ± SD (mm)
Hurricane Matthew				
Hog CAFOs and heavy storm precipitation				

0	0.97 (0.80, 1.14)	81	716	277 ± 39
1–10	1.02 (0.86, 1.18)	119	1095	279 ± 41
>10	1.05 (0.86, 1.24)	71	669	329 ± 66

Poultry CAFOs and heavy storm precipitation				

0	1.02 (0.80, 1.24)	57	431	272 ± 40
1–10	0.96 (0.82, 1.11)	124	1174	287 ± 50
>10	1.08 (0.91, 1.26)	90	875	310 ± 59

Hog and poultry CAFOs and heavy storm precipitation				

0 both	1.00 (0.79, 1.20)	50	474	272 ± 40
1–10	0.99 (0.86, 1.13)	163	1475	287 ± 50
>10 both	1.08 (0.87, 1.29)	58	531	320 ± 61

Hurricane Florence				

Hog CAFOs and heavy storm precipitation				

0	1.01 (0.89, 1.13)	95	839	531 ± 193
1–10	1.03 (0.90, 1.15)	96	1092	502 ± 166
>10	1.15 (1.04, 1.27)	86	1063	598 ± 138

Poultry CAFOs and heavy storm precipitation				

0	1.00 (0.88, 1.13)	100	813	588 ± 196
1–10	1.13 (0.98, 1.27)	67	934	509 ± 182
>10	1.06 (0.96, 1.16)	110	1247	519 ± 130

Hog and poultry CAFOs and heavy storm precipitation				

0 both	1.01 (0.88, 1.15)	80	699	571 ± 194
1–10	1.04 (0.93, 1.15)	134	1522	500 ± 174
>10 both	1.15 (1.02, 1.28)	63	773	592 ± 107

We observed very small and imprecise increases in AGI ED visit rates in areas with heavy storm precipitation and >10 poultry CAFOs after the hurricanes (Matthew: RR = 1.08, 95% CI: 0.91, 1.26; Florence: RR = 1.06, 95% CI: 0.96, 1.16, table 2). During the three-week period after Hurricane Florence, there was a 13% increase in AGI ED visit rate in areas with 1–10 poultry CAFOs and heavy hurricane precipitation (RR = 1.13, 95% CI: 1.27). In areas with heavy storm rain, >10 hog CAFOs, and >10 poultry CAFOs, we observed a 15% increase in AGI ED visit rates during the three weeks after Hurricane Florence (RR = 1.15, 95% CI: 1.02, 1.28); we did not observe a substantial increase in these areas during the three-week period after Hurricane Matthew (RR = 1.08, 95% CI: 0.87, 1.29).

In our sensitivity analyses, we observed relatively null results for various time periods ranging between 1 and 5 weeks after Hurricane Matthew in areas with heavy storm precipitation and various hog CAFO categories (figure S1). While we observed increases in AGI ED visit rates during the three- and four-week periods after Hurricane Florence in ZIP codes with heavy storm precipitation and >10 hog CAFOs, we observed decreases in AGI ED visit rates in areas with heavy storm precipitation and >0 hog CAFOs during the one-week period after/during Hurricane Florence (1–10 hog CAFOs: RR = 0.82, 95% CI: 0.63, 1.00; >10 hog CAFOs: RR = 0.81, 95% CI: 0.64, 0.98; figure S1). Upon examining ZIP codes with >20 hog CAFOs and heavy storm precipitation, we observed very slight, imprecise increases in AGI ED visit rates during the three week after Hurricanes Matthew and Florence (Matthew: RR = 1.10, 95% CI: 0.83, 1.36, *n* = 300 AGI ED visits, 37 ZIP codes, mean precipitation: 323 ± 71 mm; Florence: RR = 1.13, 95% CI: 0.97, 1.29, *n* = 441 AGI ED visits, 43 ZIP codes, mean precipitation: 625 ± 120 mm). We observed a 34% increase in AGI ED visit rate during one-week period after Hurricane Matthew in areas with heavy hurricane precipitation and >20 hog CAFOs, although this is based on only 130 AGI ED visits during this period (RR = 1.34, 94% CI: 1.06, 1.61, 37 ZIP codes, mean precipitation: 323 ± 71 mm). When using Hurricane Florence’s upper quartile of storm precipitation (325 mm) to designate heavy storm rain for Hurricane Matthew, we observed fairly similar results to our main analysis of Matthew (table S1). While we did not observe significant increases in AGI ED visit rates after Hurricane Matthew, we observed a suggestive, imprecise 17% increase in AGI ED visit rates during the two weeks after Matthew in areas with >10 hog CAFOs and >323 mm storm precipitation (RR = 1.17, 95% CI: 0.94, 1.41; table S1).

We observed similar results in our sensitivity analyses that included total number of animals as we observed in our main analyses of number of CAFOs (table S2). We observed a 16% increase in AGI ED visit rate during the three weeks during/after Florence in ZIP codes with >10 000 hogs and heavy hurricane precipitation (RR = 1.16, 95% CI: 1.05, 1.27) and no increase in areas with 0 hogs or 1–10 000 hogs. During this three-week post-Florence period, we also observed a 13% increase in ZIP codes with 1–1000 000 birds (RR = 1.13, 95% CI: 0.99, 1.28) and a 10% increase in ZIP codes with >1000 000 birds and >10 000 hogs (RR = 1.10, 95% CI: 0.98, 1.21, table S2). Additionally, the results from our sensitivity analyses that adjusted for SSOs were similar to our main analyses (table S3). Lastly, when examining the change in total ED visit rates after the hurricanes in the same ZIP codes with heavy hurricane precipitation and various levels of CAFOs, we observed no increase in total ED visit rate during the three weeks after Hurricane Florence in areas with heavy rain and 0 CAFOs, 1–10 hog CAFOs, or >10 hog CAFOs (table S2).

## 4. Discussion

In this paper, we found that areas with heavy hurricane precipitation and many hog CAFOs experienced an increased AGI ED visit rate during the three-week period after Hurricane Florence, compared to their expected AGI ED rates. We also observed an increase in AGI ED rates in areas with >20 hog CAFOs and heavy hurricane precipitation during the one-week period after Hurricane Matthew and a suggestive increase in areas with >10 hog CAFOs and extremely heavy storm precipitation during the two-week period after Matthew. This difference in timing of AGI ED visit rate increase is likely due to differences in intensity, duration, and antecedents between the storms. We did not observe an increase in AGI ED visit rate during the one- to five-week periods after the hurricanes in ZIP codes without hog CAFOs and with heavy storm precipitation, suggesting that the increase we saw after Florence in areas with heavy storm rain and many CAFOs may not be an independent effect of the hurricane. We also observed no increase in overall ED visit rate in these areas during the three weeks after Hurricane Florence, further suggesting that the presence of hog CAFOs in these communities may have led to the increased AGI incidence. Areas with many hog CAFOs and heavy storm precipitation were more vulnerable in terms of SES, disability, and availability of transportation than areas with heavy storm precipitation and no hog CAFOs.

**Figure 3.**
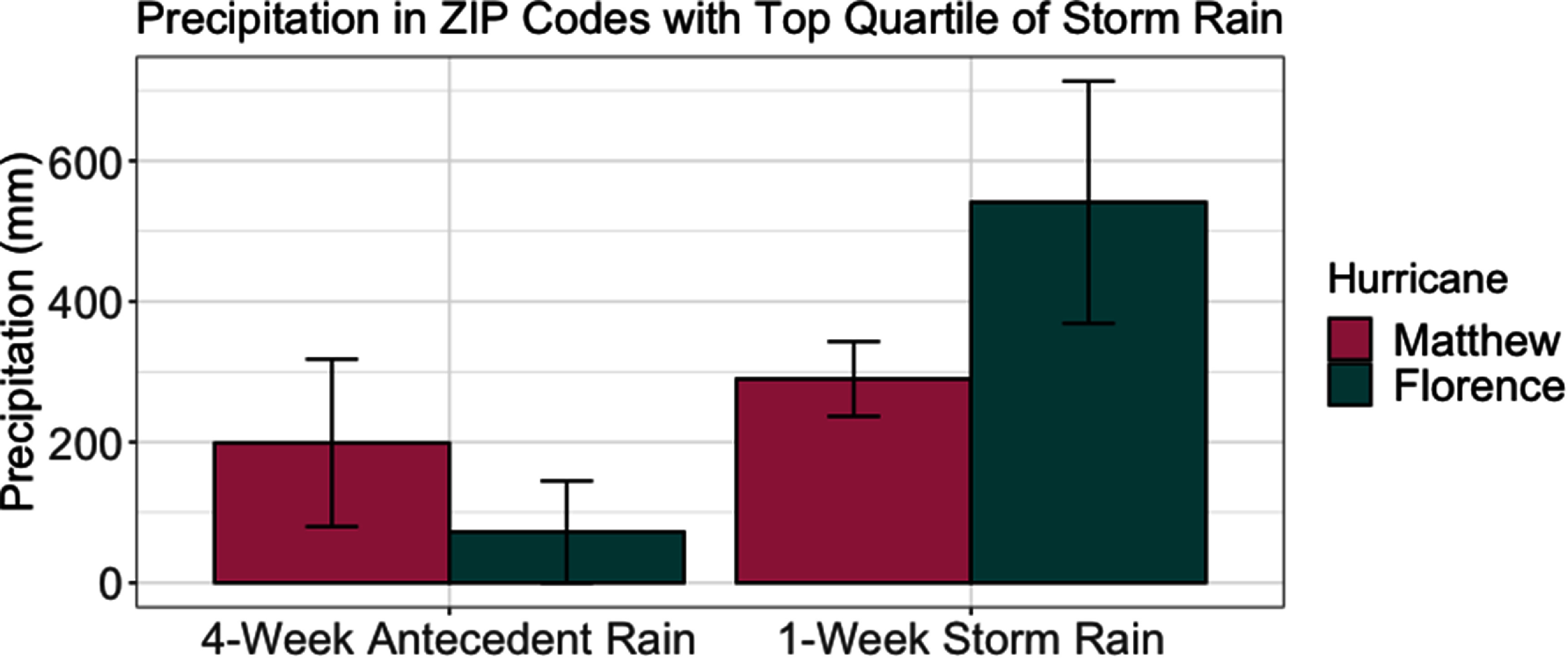
Precipitation in ZIP codes with the top quartile of storm rain during the four weeks before and the one week during/after Hurricanes Matthew and Florence.

Although Matthew and Florence struck fairly similar areas of NC (see figures 1 and 2), Florence dropped substantially more water on NC (maximum rain from Matthew: 19 inches; maximum rain from Florence: 36 in.; see figure 3) [32, 33]. Also, several heavy rain events preceded Hurricane Matthew, while Hurricane Florence was preceded by a dry period. Hurricane Hermine struck NC five weeks before Hurricane Matthew, dropping up to 13 in. of rain, and severe heavy rain events, dropping up to 10 in. of rain, occurred just nine days before Matthew [44]. These heavy rain events prior to Hurricane Matthew may explain why we saw an immediate increase in AGI ED rate, as river levels were already relatively high and most of Matthew’s precipitation fell on one day. Hurricane Florence was a slow-moving hurricane that stalled over NC, with parts of the state receiving up to 36 inches of rain [34]. While many rivers crested within 4 d of Hurricane Florence’s landfall, some crested 9 d later, which may explain the delayed increase in AGI ED visit rate until 3 weeks after the storm [52]. The decrease in AGI ED visit rates in areas with heavy storm precipitation and >0 hog CAFOs during the one-week period during/after Hurricane Florence is somewhat unexpected; however, this decrease could be due to the difficulty of rural residents in traveling to EDs during the week of Hurricane Florence because of the weeklong flooding. The differences we see during the first week during/after Hurricanes Matthew and Florence are likely because Florence caused much more destruction, was a slower and longer-lasting storm, and caused more people to evacuate than Hurricane Matthew [32, 33]. Previous studies have shown all hurricanes affect the environment and water quality differently; these differences can also be from the hurricanes’ direction after landfall, which also differed between Matthew and Florence [37, 40].

While the strongest increase in AGI ED visit rates occurred in the areas with heavy storm precipitation and >10 *hog* CAFOs during the three weeks after Florence, we also observed a suggestive increase in areas with 1–10 and >10 *poultry* CAFOs and heavy Florence rain, although these areas also contain hog CAFOs (451 hog CAFOs in ZIP codes with 1–10 poultry CAFOs and heavy storm rain, 1961 hog CAFOs in ZIP codes with >10 poultry CAFOs and heavy storm rain). When examining ZIP codes with both poultry and hog CAFOs, the results were similar to what we observed when examining just hog CAFOs. It is difficult to disentangle the effects of poultry and hog CAFOs because they are so commonly co-located in flood-prone eastern NC. Central and western NC contain poultry CAFOs without nearby hog CAFOs, but these areas received less rain during the hurricanes, making direct comparison difficult. Previous analyses of this NC ED data found that ZIP codes with high hog CAFO exposure had an 11% higher AGI ED rate than control areas and that areas with both poultry and hog CAFO exposure had a 52% higher AGI ED rate [27]. However, in this current paper, which considers both CAFOs and hurricane precipitation, we did not observe a higher AGI rate in areas with both poultry and hog CAFOs than in areas with just hog CAFOs.

Several of the findings in this paper are confirmed by other studies. Heavy rain and flooding have been linked to an increase in gastrointestinal illness rate, even in areas without CAFOs, because sewer overflows, overwhelmed municipal water systems, and damaged septic systems increase the spread of pathogens [41, 45, 46, 53, 54]. The results from our sensitivity analysis that incorporated SSO data were similar to our main results, highlighting that SSOs were not driving the increase in AGI ED visit rate we observed after Hurricane Florence. Additionally, areas with >10 hog CAFOs did not have a higher total volume of SSOs during the week after the hurricanes struck NC than areas with 1–10 hog CAFOs (table S3). While some studies have observed an increase in AGI rate during the 0–5 d after flooding [45, 54], others have seen the increase in AGI rate occur 7–30 d after flooding [41, 53, 55]. The null result this study observed in ZIP codes with no hog CAFOs and heavy storm precipitation was somewhat unexpected, but some studies have also observed no association and our results indicate that nearby environmental exposures may play a large role in the relationship between heavy hurricane precipitation and AGI rate [56, 57]. Although prior analyses of this NC ED data found a small increase in rate of AGI ED visit rate during the three weeks after Hurricanes Matthew and Florence in areas with severe flooding, those analyses included all heavily flooded areas and did not consider other environmental co-exposures such as hog CAFOs [41]. A recent study found that ED visits decreased in flooded census tracts during the month following Hurricane Harvey (2017) and that the decrease was smaller in areas with moderate, high, and very high vulnerability [58]. Their results suggest that flood survivors with inadequate housing and transportation used EDs for healthcare during and after the flooding more than they normally did. One study found that hurricane-related ED visits for medication refills in NC were higher during the weeks after Hurricane Florence than before Florence, indicating that many residents use EDs to obtain medication when pharmacies are closed after large hurricanes [59]. Our study highlights how residents who experienced heavy storm rain and were proximate to many hog CAFOs had more underlying social vulnerability than the state vulnerability average. Social vulnerabilities may affect ED usage and disaster vulnerability, and the socially vulnerable are often more likely to be exposed to harmful environmental exposures including CAFOs.

Our results that areas with heavy hurricane rain and hog CAFOs have a higher proportion of Black and American Indian residents than the NC state average have also been shown in other studies over at least two decades. In 1999, Hurricane Floyd caused five hog lagoons to breach and at least 50 lagoons to flood in NC [3]. Numerous lagoons suffered structural damage. Wing *et al* found that, according to satellite images from Hurricane Floyd, African Americans were more likely than white people to live in areas with flooded hog CAFOs in NC [8]. Another study estimated that flooding affected 303 hog lagoons after Hurricane Matthew and 287 hog lagoons after Hurricane Florence (with affected by flooding defined as hog lagoons that flooded or were within 60 m of detected flooding) [52]. These same analyses estimate that 299 permitted wastewater treatment plants (41% of wastewater treatment plants in the NC study area) were affected by Hurricane Florence flooding and 239 (33%) were affected by Matthew [52]. Studies found elevated concentrations of *E. coli,* as well as both human and swine-associated fecal markers, in surface water after Hurricanes Matthew and Florence, suggesting that these hurricanes spread fecal waste [39, 60, 61]. Researchers also observed *Salmonella typhimurium* in water samples near hog CAFOs after Hurricane Florence [62].

Although there is a rich literature on the effects hurricanes have on water quality, few papers investigated health outcomes associated with this flooding. Setzer and Domino examined the health effects of flooded hog CAFOs in NC using Medicaid outpatient data to assess whether Hurricane Floyd was associated with increased waterborne disease-related outpatient visits in eastern NC [63]. They examined counties with high concentrations of hogs (defined as >1000 hogs) and classified the counties on the impact of Hurricane Floyd measured by the Federal Emergency Management Agency’s (FEMA) assessment of the socioeconomic impact of Floyd (severe, moderate, minor, not affected). The study is somewhat limited by these definitions, as FEMA’s designation of hurricane impact is over the entire county and does not assess which hog CAFOs were affected by the heaviest precipitation. Using difference-in-differences, they found an increase in visits for ill-defined intestinal infections in severely and moderately affected counties, compared to unaffected counties. However, the study did not draw any conclusions regarding the combined effect of hurricane flooding and hog CAFOs on gastrointestinal illness, partly because their study did not include any counties that were affected by Floyd that did not have a high concentration of hogs—possibly because most counties severely harmed by Floyd contained hog CAFOs [63].

While other studies have not examined the health effects of hurricane precipitation in combination with hog CAFOs, several studies have found increased concentrations of *E. coli, Clostridium*, and *Giardia* (which can cause AGI) in surface water and wells after heavy rain events, with stronger associations in areas with swine manure [14, 64]. Similarly, Febriani *et al* observed an association between high precipitation periods in the fall season and increased AGI risk three weeks later; they also found industrial farming and season to modify the association between cumulative precipitation and AGI four weeks later [65]. These papers and others highlight that hog CAFOs are associated with increased AGI even during non-hurricane periods. Hog waste from lagoons is regularly sprayed onto nearby fields in NC, leading to elevated levels of nitrate, ammonium, phosphorus, and fecal coliform in surface water near poultry and hog CAFOs in NC [11]. Runoff from fields with recent hog manure application has been found to have higher concentrations of *E. coli* compared to control fields; thus, hog CAFOs can pollute surface and groundwater even if manure lagoons do not spill [14]. In a previous paper using the same NC ED data as this study, our study team found that the positive association between high hog exposure and AGI ED visit rate was stronger when a heavy precipitation event (>99th percentile of daily precipitation, >2.4 inches) had occurred within the previous week than when the previous week had been dry [27]. That study supports this paper’s conclusions that exposure to both heavy hurricane precipitation and many hog CAFOs appears to increase AGI ED rate.

This study’s strengths include using interrupted time series to compare ZIP codes to themselves over time as well as the examination of two hurricanes that struck the same general areas only two years apart. Comparing areas to themselves over time allows control for known and unknown time-invariant confounders, like demographics and constant environmental exposures [48]. We also incorporated data on total number of hogs and birds as well as SSOs in sensitivity analyses to highlight the robustness of our results. We observed elevated AGI ED visit rates after Florence in ZIP codes with >10 hog CAFOs as in ZIP codes with >10 000 hogs; our results were similar when we measured CAFO exposure by number of CAFOs or by number of animals. Our study was limited by our inability to obtain information as to how the heavy storm precipitation compromised hog CAFOs and hog lagoons, as some lagoons breached, others experienced significant structural damage, and others only flooded. These different impacts of heavy precipitation on hog lagoons are likely to have large effects on the amount of hog waste and fecal bacteria that subsequently contaminate waterways. Because this information was unavailable, we examined the effect of heavy precipitation as a surrogate measure. This study is also limited by the ZIP code-level ED data. However, this ZIP code-level analysis is an improvement in geographic granularity over other studies that examined this question at a county level.

Our analyses were also limited because the demographics of the areas with many hog CAFOs and heavy rain during hurricanes were quite different from those of areas without hog CAFOs. We compared AGI ED rates in ZIP codes after hurricanes to their expected AGI ED rates had the hurricanes not occurred because appropriate control areas could not be created. Our prior efforts to make hog CAFO and hog CAFO-free control areas comparable via weighting were unsuccessful because of marked sociodemographic differences between these areas (not shown). Because of these limitations, we are unable to make causal statements from our results. The differences in demographics and social vulnerability between the categories of ZIP codes of heavy storm rain with 0, 1–10, and >10 hog CAFOs could affect their ED usage patterns and how these populations responded to and recovered from the hurricanes. Thus, caution is required when comparing results between these CAFO count categories. Nevertheless, our findings that there was no increase in *total* ED visit rate during the three weeks after Hurricane Florence in areas with heavy storm rain and >10 hog CAFOs support our conclusion that the observed increase in AGI ED visits was related to the presence of the hog CAFOs. The unequal distribution and simultaneous concentration of hurricane-prone areas, hog CAFOs, and communities of color in ‘sacrifice zones’ can cause structural confounding issues that make causal analysis difficult [66]. The high storm precipitation ZIP codes with >10 hog CAFOs received more rain, on average, than high precipitation ZIP codes with 0 hog CAFOs (table 2), highlighting that hog CAFOs are located in areas that receive an especially large amount of rain during hurricanes. However, this also makes it difficult to identify hog CAFOs as the causal agent. Most areas in NC that experienced heavy precipitation and flooding from these hurricanes have many hog CAFOs (except for the coast, which has very different demographics) and most unflooded areas have few or no hog CAFOs. This highlights an important environmental justice and climate justice issue, that flooding and related environmental health problems disproportionately harm low-income residents and people of color, who are also disproportionately harmed by hog CAFOs in NC. Historically, several Black towns, like Princeville, NC, were established in flood plains, as this was some of the only land available to Black people [67]. Additionally, a recent study found that the current legal NC floodplain underestimates the impacts of flooding on areas with high proportions of older adults, disabled individuals, unemployment, and mobile homes [52]. Existing social vulnerabilities and environmental injustices often contribute to disaster vulnerabilities [68].

## 5. Conclusions

Hurricanes will continue to hit NC and hog lagoons will continue to flood and spread pathogens despite wide discussion of the effects of flooded and damaged lagoons and the ban on building new lagoons in the 100 year floodplain [3]. The co-occurrence of hog CAFOs in communities of color and climate change impacting those same communities through hurricanes doubly harms these communities now and in the future. Over the last few decades, NC’s regulation of hog CAFOs has changed very little about these disproportionate exposures; instead, risks have increased over time as the industrial poultry industry has expanded in many of the same areas, and hurricanes have become more frequent and intense [69, 70]. Although the NC Swine General Permit provides some protection to the environment and nearby communities under usual conditions, this study and others suggest that the protection may be inadequate at preventing health problems resulting from the spread of hog waste during hurricanes and other heavy precipitation events. In addition to the human health effects from flooding at CAFOs, tens of thousands of hogs and poultry drowned during Hurricanes Floyd, Matthew, and Florence, and lagoon breaches during these storms killed many fish and caused algae blooms [71, 72]. While this paper focuses on AGI possibly caused by fecal bacteria, hog manure also contains nitrates, heavy metals, and antibiotic residues that also harm the environment and may adversely affect human health [73–77]. Hurricanes and heavy precipitation events are expected to continue increasing in frequency and intensity in the coming years because of climate change [78]. The intersection of CAFOs and flooding has created complex environmental and climate justice issues that are exacerbated during every hurricane. Areas with hog CAFOs and frequent hurricane flooding in NC contain vulnerable communities that may be at increased risk for AGI after hurricanes. Disaster preparedness and response must consider both environmental and social vulnerabilities to improve health and reduce health disparities in NC.

## Data Availability

The data cannot be made publicly available upon publication because they are owned by a third party and the terms of use prevent public distribution. The data that support the findings of this study are available upon reasonable request from the authors.
